# Eaten alive: cannibalism is enhanced by parasites

**DOI:** 10.1098/rsos.140369

**Published:** 2015-03-18

**Authors:** Mandy Bunke, Mhairi E. Alexander, Jaimie T. A. Dick, Melanie J. Hatcher, Rachel Paterson, Alison M. Dunn

**Affiliations:** 1 School of Biology, University of Leeds, Leeds LS2 9JT, UK; 2 Centre for Invasion Biology, Department of Botany and Zoology, Stellenbosch University, Matieland 7602, South Africa; 3 Institute for Global Food Security, School of Biological Sciences, Queen's University Belfast BT9 7BL, , UK

**Keywords:** parasitism, cannibalism, amphipod, behaviour

## Abstract

Cannibalism is ubiquitous in nature and especially pervasive in consumers with stage-specific resource utilization in resource-limited environments. Cannibalism is thus influential in the structure and functioning of biological communities. Parasites are also pervasive in nature and, we hypothesize, might affect cannibalism since infection can alter host foraging behaviour. We investigated the effects of a common parasite, the microsporidian *Pleistophora mulleri*, on the cannibalism rate of its host, the freshwater amphipod *Gammarus duebeni celticus*. Parasitic infection increased the rate of cannibalism by adults towards uninfected juvenile conspecifics, as measured by adult functional responses, that is, the rate of resource uptake as a function of resource density. This may reflect the increased metabolic requirements of the host as driven by the parasite. Furthermore, when presented with a choice, uninfected adults preferred to cannibalize uninfected rather than infected juvenile conspecifics, probably reflecting selection pressure to avoid the risk of parasite acquisition. By contrast, infected adults were indiscriminate with respect to infection status of their victims, probably owing to metabolic costs of infection and the lack of risk as the cannibals were already infected. Thus parasitism, by enhancing cannibalism rates, may have previously unrecognized effects on stage structure and population dynamics for cannibalistic species and may also act as a selective pressure leading to changes in resource use.

## 2. Introduction

Cannibalism has been recorded in more than 3000 species [1–4] and may be influential at the levels of individuals, populations and communities. It is especially common in stage-structured populations where generations overlap in time and space [2,5]. Direct individual benefits of cannibalism include increased growth and survival [3], while indirect positive effects include the elimination of competitors [6]. Cannibalism may also enhance population persistence when resources are limited; for example, cannibalism may function as a ‘lifeboat mechanism’ whereby cannibalistic adults have access to resources and energy accrued by the cannibalized juveniles [7]. There are, however, a number of costs associated with cannibalism, including the acquisition of parasites via consumption of infected conspecifics [8].

Parasitism is also pervasive in nature [9] and influences a number of intra- and interspecific interactions, including competition and predation, through both density- and trait-mediated effects [9–11]. In particular, parasites can modify the rate of predatory interactions [12,13] as well as alter the vulnerability of infected hosts to predation [9,14]. Parasitism, we propose, may therefore also be an important determinant in cannibalistic interactions with implications for population structure and community dynamics. This may be evidenced through changes in host behaviour as a result of metabolic costs [12], parasite manipulation to increase transmission likelihood [15–17], or can reflect selection on hosts to avoid costs of infection [17].

The microsporidian parasite *Pleistophora mulleri* is specific to the amphipod *Gammarus duebeni celticus*. It has a prevalence of up to 90% and can alter predation hierarchies among species [13] with both parasitized and unparasitized individuals occurring in close proximity to one another [18]. There is a large body of evidence that indicates *G. d. celticus* commonly engages in cannibalism in the field [19]. In addition, the only known route for the transmission of the microsporidian is cannibalism, providing further evidence of field cannibalism [20]. Therefore, as the parasite is transmitted orally, with an efficiency rate of 23% [20] and, as cannibalism in this species is common, it imparts a risk of infection of *P. mulleri* [20]. As such, parasite mediation of cannibalism may occur with important implications for host populations. We therefore investigated whether the cannibalistic rate and preferences of *G. duebeni celticus* are affected by infection with *P. mulleri*.

We used a ‘functional response’ approach (FR; resource uptake as a function of resource density), which can inform on consumer impacts on resource populations [21]. First, we investigated the impact of parasitism on cannibalistic propensity by deriving FRs for individuals with and without the parasite. Second, we used an intraspecific prey choice experiment to test whether infected and uninfected *G. d. celticus* showed any preferences with respect to the infection status of juvenile conspecific victims.

## 3. Material and methods

Adult male and juvenile *G. d. celticus* were collected from Downhill River, County Antrim, Northern Ireland (55.166674 N, 6.8201185 W) in November 2010 and April 2011. No permissions are required for this sampling activity. Males were selected for experiments owing to the wide variation in female cannibalism that can occur due to factors relating to egg and embryo brooding [22]. Parasite status was determined by the presence/absence of *P. mulleri* spore mass visible through the exoskeleton (status confirmed by later dissection) and parasitized individuals all had visible infection of one to two segments [23]. Animals were separated according to infection status and maintained in aquaria with water and leaf material from their source at 12° C and a 12 L : 12 D cycle.

For FR experiments, we selected similar-sized infected and uninfected adult male *G. d. celticus* (body mass (*mg*)±*s*.*e*., infected 52.57±1.49, uninfected 50.90±1.23; two-sample *t*-test, *t*=0.86, *p*>0.05). We presented single infected and uninfected males (starved for 48 h) with uninfected juveniles (4–6 mm body length) at seven juvenile densities (2, 4, 6, 8, 10, 15, 20; *n*=3 per density) in plastic dishes (8 cm diameter) containing 200 ml of aerated water from the amphipod source river. The densities of juvenile prey used were informed by previous FR studies on gammarids in combination with known densities from the wild that are hypervariable and can reach several thousand per square metre [24]. Controls were three replicates of each juvenile density without adults. Replicates were initiated at 17.00 h and prey consumption was examined after 40 h.

Mean number of juveniles killed was examined with respect to adult infection status and juvenile density using generalized linear models (GLMs) with quasi-poison error distribution in R v. 3.0.1 that were simplified via a step-deletion process. We determined FR types using logistic regression of the proportion of prey consumed against initial prey density [25] and modelled FRs using the Rogers' random predator equation for a Type II FR, which accounts for non-replacement of prey as they are consumed [26]. FR data were bootstrapped (*n*=15) and the parameters attack rate *a*, handling time *h* and maximum feeding rate 1/*hT* (*T*=experimental time) compared using GLMs.

Preferences of infected and uninfected adults for cannibalism of infected versus uninfected juveniles were investigated by presenting adult males (*n*=30 uninfected and 30 infected individuals; sizes as above; starved for 72 h) with a choice between an infected and uninfected juvenile individual (6 mm body length; matched by weight) in plastic dishes (10 cm diameter, 150 ml volume). Trials began from the addition of the prey and were terminated when a prey item had been selected. Prey choice by the adults with respect to juvenile infection status was determined using *χ*^2^-tests.

## 4. Results

**Figure 1.**
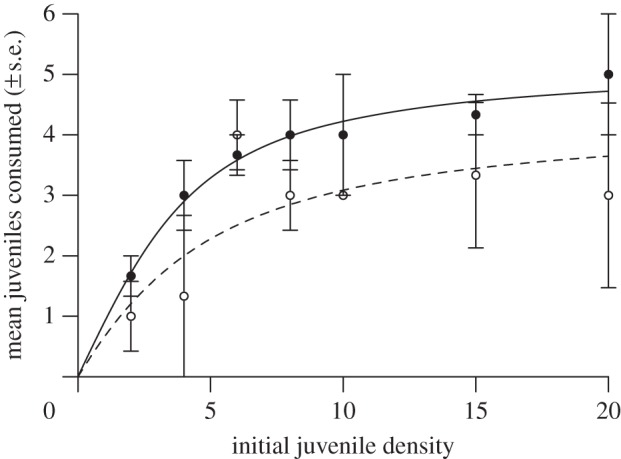
FRs of infected (filled circles, solid line) and uninfected (open circles, dashed line) *Gammarus duebeni celticus* adults towards juvenile conspecific prey. Lines are modelled by the Rogers' random predator equation for a Type II response. Data points are mean numbers of juveniles consumed at each density±s.e.

**Figure 2.**
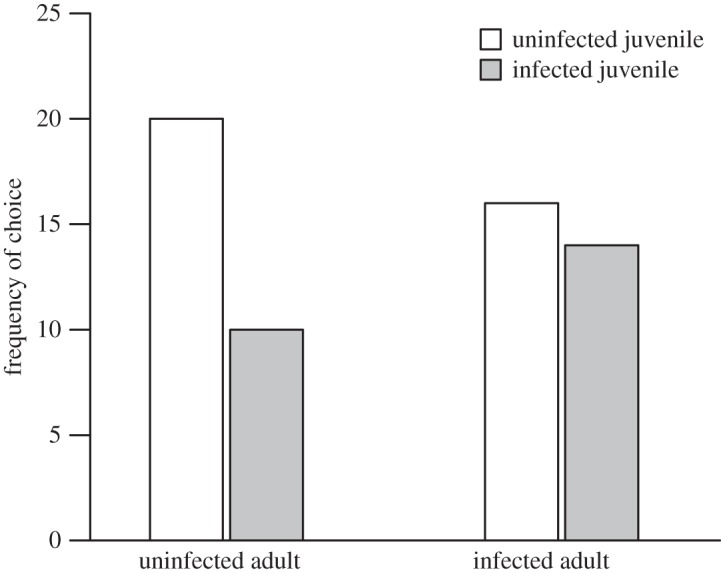
The frequency of consumption of uninfected versus infected juveniles by uninfected and infected adult *Gammarus duebeni celticus*.

Control juvenile *G. d. celticus* survival was high (99.5%), thus experimental deaths were attributed to cannibalism by adults. This was further evidenced through observation and amphipod body parts littering the aquarium floor. Significantly more juveniles were eaten by infected than uninfected adults (*F*_1,40_=5.03, *p*<0.05; figure 1) and both FRs were found to follow a Type II curve (figure 1). Infected adults had significantly greater attack rates *a* (*t*=5.87, *p*<0.001) and significantly lower handling times *h* (*t*=3.67, *p*<0.01). This translated into significantly higher maximum feeding rates (1/*hT*) (*t*=2.71, *p*<0.05) in comparison to uninfected individuals (figure 1). Uninfected adults more frequently consumed uninfected than infected juveniles ($\chi_{1}^{2}=4.8$, *p*<0.0285; figure 2), whereas infected adults showed no preference ($\chi_{1}^{2}=1.333$, *p*>0.05; figure 2).

## 5. Discussion

Although the role of parasitism in interspecific predator–prey interactions has been studied in a number of systems [12,13], the influence of parasites in mediating cannibalism has received far less attention, despite cannibalism and parasitism both being widespread and pervasive in natural communities [3,9]. Parasites may affect cannibalism since they have been shown to affect foraging behaviour, both increasing and decreasing host consumption of resources, with potential implications for population dynamics and community structure in such taxa [12].

Here, the FR of the amphipod *G. d. celticus* infected with the microsporidian parasite *P. mulleri* towards juvenile (uninfected) conspecific prey was significantly higher in comparison to uninfected adults. Furthermore, infected amphipods had significantly greater attack rates, decreased handling times and hence heightened maximum feeding rates, demonstrating that infected amphipods are more efficient than their uninfected counterparts at cannibalizing juveniles. This probably reflects the metabolic burden imposed by the parasite, leading to higher feeding rates [12]. That infected individuals are such efficient foragers is despite the fact that this parasite degrades host tissue and substantially debilitates its host [27].

The preferential consumption of uninfected juveniles by uninfected adults probably reflects selection for avoiding cannibalizing infected juveniles and therefore reducing the risk of parasite acquisition [8,28]. On the other hand, infected adults showed no such discrimination. One explanation for this lack of discrimination may be that immune priming or immune upregulation protects infected individuals from further infection [29]. However, Terry *et al.* [27] found no evidence of encapsulation or other immune responses in *P. mulleri* infected hosts. Rather, we suggest that the lack of discrimination in cannibalism of infected versus uninfected juveniles by infected adults again reflects the metabolic burden of infection whereby parasitized individuals cannot afford to be as selective in what prey they consume. Furthermore, as they are already infected with the parasite, there is no advantage to avoiding infection risk by preferentially consuming uninfected prey.

Overall, we show that infection of *G. d. celticus* with the parasite *P. mulleri* altered cannibalism rates and feeding preferences on juvenile conspecific victims. This in turn may increase the rate of juvenile mortality (over and above conventional virulence effects), which could lead to changes in population stage structure and density [5,11,30]. Furthermore, this interplay between cannibalism and parasitism could have powerful impacts on population and community resilience in changing environments, whereby cannibalism becomes an important mechanism in preserving populations [7], although in the wild, population outcomes will also depend on the relative importance of interspecific predation and cannibalism. Cannibalism and intraguild predation co-occur frequently in a broad range of systems [1,5] and the balance of these intra- versus interspecific interactions is key to species coexistence and replacement patterns [31]. Parasites are also recognized as having important indirect and pervasive effects on communities and ecosystems, often owing to their density and trait-mediated indirect effects on species that interact with their hosts [32]. Further exploration of parasite-modified cannibalism thus has potential to further understand and predict population dynamics and community processes.

## Supplementary Material

Bunkeetal_OpenScience_data.xlsx Data for experiments detailed in MS

## Data accessibility

The datasets supporting this article have been uploaded as part of the electronic supplementary material.
